# ATF4-mediated histone deacetylase HDAC1 promotes the progression of acute pancreatitis

**DOI:** 10.1038/s41419-020-03296-x

**Published:** 2021-01-04

**Authors:** Xiaofeng Deng, Yu He, Xiongying Miao, Bo Yu

**Affiliations:** 1 Department of General Surgery, the Second Xiangya Hospital of Central South University, Changsha, 410000 P. R. China; 2 Department of Radiology, the Second Xiangya Hospital of Central South University, Changsha, 410000 P. R. China; 3 Department of Critical Care Medicine, the Second Xiangya Hospital of Central South University, Changsha, 410000 P. R. China

**Keywords:** Cell biology, Cell death

## Abstract

Acute pancreatitis (AP), an acute inflammatory process, can be difficult to diagnose. Activating transcription factor 4 (ATF4) has been reported to participate in the pathogenesis of AP. Additionally, histone deacetylases (HDACs) are shown to be closely related to the development of a variety of diseases, including inflammation disease. In our study, we tried to highlight the role of ATF4 in AP through regulation of HDAC1. Firstly, we validated the effect of ATF4 on pancreatic acinar cell proliferation, apoptosis, and inflammation through in vitro experiments on cellular models of caerulein-induced AP. Next, we examined the correlation between ATF4 and HDAC1, and between HDAC1 with neutral endopeptidase (NEP) and kruppel-like factor 4 (KLF4). Finally, the regulatory role of ATF4 in AP was further assessed by determination of pathological conditions, biochemical indicators and inflammation through in vivo experiments on caerulein-induced AP mouse models. After AP induction, highly expressed ATF4 was observed, and silencing ATF4 could promote pancreatic acinar cell proliferation and inhibit apoptosis. ATF4 could bind to the HDAC1 promoter and upregulate its expression in AP. Moreover, HDAC1 could increase KLF4 expression by inhibiting NEP expression. Functionally, silencing ATF4 could suppress AP through regulation of NEP-mediated KLF4 *via* downregulation of HDAC1. Above all, our study uncovered the promotive role of ATF4 in AP through upregulation of HDAC1.

## Introduction

Acute pancreatitis (AP), an inflammatory disease, is the main cause of hospitalization for gastrointestinal disorders in the United States and many other countries^1^. The rising incidence of this disease might further augment the frequency of disorders in several systems (such as endocrine, exocrine, and aberrant bone metabolism) long after clinical treatment of pancreatitis^2^. Several promising strategies have emerged as novel therapeutic options for the early management of AP, such as the application of enteral nutrition and antibiotics, haemofiltration and protease inhibitors^3^. The pancreatic acinar cells are a crucial cell source for the synthesis, delivery, storage, and secretion of digestive enzymes while the dysfunction of those cells initiate and propagate inflammation whereby stimulating pancreatitis^4^. No curative therapy has been currently developed for AP. Early laparotomy is applied for the removal of the necrotic tissues in the patients with necrotising pancreatitis but is related to high mortality probably due to the unbearable burden to surgical trauma in severe cases^5^. Unfolded protein response and autophagy exert cytoprotective role by reversing sustained endoplasmic reticulum (ER) stress, and inhibiting apoptosis and necrosis. Characterization of these pathways contributes to the development of novel molecular targets for prospective therapeutic trials^6^.

Transcription factors are key mediators of DNA-binding transcription and chromatin via recognizing specific DNA sequences and function in diverse human physiological events and disorders^7^. Activating transcription factor 4 (ATF4) is an unfolded protein response component that is induced downstream of such metabolic stresses as ER stress and is observed to be upregulated in chronic pancreatitis^8^. Also, ATF4 has been previously documented to be one of deregulated genes in the asparaginase-associated pancreatitis^9^. Intriguingly, ATF4 positively regulates the expression of histone deacetylase 1 (HDAC1)^10^, which is associated with regulation of NOD-like receptor protein 3 inflammasomes and immune cellular infiltration in pancreas^11^. HDACs are a group of enzymes that reverse histone acetylation and their inhibitors have therapeutic value for inflammatory disorders^12^. HDACs can affect the inflammatory response in AP with the recruitment of histone acetyltransferases^13^. HDAC1 induces histone deacetylation in the neutral endopeptidase (NEP) promoter, resulting in inhibition of NEP^14^. NEP was first identified as a neuropeptide-degrading enzyme in the pancreas in 1992^15^. In addition, NEP is proposed as a protective biomarker in caerulein-induced AP^16^. Kruppel-like factor 4 (KLF4) is a transcription factor which controls many cellular processes such as cell proliferation and differentiation, and can be negatively regulated by NEP in pulmonary artery smooth muscle cells^17^. Based on the above findings, we further focused on the functionality of ATF4, HDAC1, NEP, and KLF4 in the pathogenesis of AP as well as their interaction in the regulation of pancreatic acinar cells.

## Materials and methods

### Ethics statement

Animal experiment protocols were approved by the Second Xiangya Hospital of Central South University. All animal experiments were performed in accordance with the Guide for the Care and Use of Laboratory animals published by the US National Institutes of Health. Appropriate measures were taken to minimize the use of animals as well as their suffering.

### Microarray-based gene expression analysis

The microarray dataset GSE3644 in Gene Expression Omnibus (GEO) database (https://www.ncbi.nlm.nih.gov/geo/) was analyzed by “limma” package of R language programming with |logFoldChange| > 1.5, and *p* < 0.01 as threshold. The microarray dataset included 6 normal samples and 6 pancreatitis samples. Human transcription factor names were obtained through Cistrome (http://cistrome.org), and important transcription factors were obtained by comparing differential genes with transcription factors. GeneMANIA (http://genemania.org) was used to predict genes related to important transcription factors, followed by construction of protein–protein interaction (PPI) network. Cytoscape (https://cytoscape.org) was applied to plot and calculate core levels and scores to determine the most critical transcription factors, and the R language was used to obtain the expression data in the microarray dataset GSE3644 to draw the box plots of the key transcription factors to determine their expression trends. The site of transcription factors was obtained through JASPAR (http://jaspar.genereg.net), and the downstream pathways of key transcription factors were identified through existing literature. MEM (https://biit.cs.ut.ee/mem/index.cgi) was used for co-expression analysis to verify the feasibility of the downstream pathway, and then GeneMANIA was applied to predict the relevant genes of the most downstream genes, followed by construction of a PPI network. Images were constructed by Cytoscape. KEGG enrichment analysis was performed by KOBAS (http://kobas.cbi.pku.edu.cn) to verify its relationship with pancreatitis.

### AP mouse model

A total of 60 C57BL/6 J male mice (6 weeks, 20–25 g) was enrolled and were kept for a week before the experiment to adapt to the new conditions, with a 12-h light/dark cycle. AP mouse model was established as previously described^18^. Briefly, AP was induced by intraperitoneal injection of 50 mg/kg cerulein (7 doses hourly). In the treatment group, corresponding lentivirus (sh-ATF4, oe-KLF4 or corresponding controls) was injected 5 days before AP induction. The control mice received normal saline hourly. Twenty-four h after AP induction, mice were euthanized, blood samples were collected and pancreases were removed and frozen in liquid nitrogen immediately. Blood samples were used to determine peroxidase, serum amylase, and lipase activities.

### Enzyme-linked immunosorbent assay (ELISA)

Serum or cell supernatant was taken to measure the levels of tumor necrosis factor-α (TNF-α) (JLC3924), interleukin-1β (IL-1β) (JLC3580), IL-6 (JLC3601), and IL-10 (JLC3554) by ELISA (Shanghai Jingkang Biological Engineering Co., Ltd., Shanghai, China) according to the instructions.

### Hematoxylin eosin (H&E) staining

A small portion of the pancreas was selected, fixed in buffered formalin (Sigma-Aldrich) with 10% neutral solution, dehydrated by ethanol and embedded in paraffin for routine histological examination. The sample was stained with H&E and examined by light microscope inspection Nikon Eclipse 80i microscope. Two pathologists scored edema, inflammation, hemorrhage, and necrosis of pancreatic tissues from 0 to 4 under 20 random high-power microscopes based on the histological scoring criteria of Kusske et al.^19^. The final scores for each group were then added up.

### Immunohistochemistry

Paraffin sections of pancreatic tissues from each group were dewaxed, dehydrated with alcohol gradient, washed with tap water for 2 min, treated with H_2_O_2_ containing 3% methanol for 20 min, washed with distilled water for 2 min, and washed with 0.1 M phosphate buffer saline (PBS) for 3 min followed by antigen retrieval. The sections were added with normal goat serum blocking solution (C-0005, Shanghai Haoran Biological Technology Co., Ltd., Shanghai, China) at 4 °C overnight, added with primary antibody to ATF4 (ab23760, 1:250, Abcam, Cambridge, UK) at 4 °C overnight, and then added with goat anti-rabbit immunoglobulin G (IgG) secondary antibody at 37 °C for 20 min. Next, the sample was added with horseradish peroxidase-labeled streptavidin protein working solution (0343-10000U, Beijing Yimo biological technology Co., Ltd., Beijing, China) at 37 °C for 20 min. After that, the sample was developed by 3,3′-diaminobenzidine (ST033, Guangzhou Weijia technology Co., Ltd., Guangdong, China), stained by hematoxylin (PT001, Shanghai Bogoo Biotechnology Co., Ltd., Shanghai, China) for 1 min, returned to blue color by 1% ammonia, dehydrated by a certain concentration of alcohol, cleared by xylene, mounted by neutral resin and observed under a microscope.

### Western blot assay

Radio Immunoprecipitation Assay lysis buffer (BOSTER Biological Technology Co., Ltd., Wuhan, China) containing protease inhibitor was used to extract the total protein. A bicinchoninic acid kit was applied to measure the total protein concentration. Protein was subjected to sodium dodecyl sulfate-polyacrylamide gel electrophoresis and transferred onto the polyvinylidene fluoride membrane, which was then blocked with 5% skim milk powder for 1 h at room temperature and probed at 4 °C overnight with the diluted primary antibodies to ATF4 (ab23760, 1:1000, Abcam), HDAC1 (ab7028, 1:1000, Abcam), NEP (ab227195, 1:2000, Abcam), KLF4 (ab215036, 1:1500, Abcam), cleaved-caspase3 (ab49822, 1:500, Abcam), B-cell lymphoma-2 (Bcl-2) (ab185002, 1:500, Abcam), Bcl-2 associated protein X (Bax) (ab32503, 1:500, Abcam), phosphorylated-Protein kinase R (PKR)-like endoplasmic reticulum kinase (p-PERK) (#3179, 1:1000, Cell Signaling Technology, USA), phosphorylated-translation eukaryotic initiation factor 2α (p-eIF2-α) (#3398, 1:1000, Cell Signaling Technology) and C/EBP homologous protein (CHOP) (#5554, 1:1000, Cell Signaling Technology). The membrane was washed for 3 times with Tris-Buffered Saline Tween-20, and re-probed with horseradish peroxidase-labeled secondary antibody of goat anti-rabbit antibody (ab205719, 1:2000, Abcam) for 1 h. Subsequently, an enhanced chemiluminescence working solution (EMD Millipore, Billerica, MA) was taken for color visualization, followed by photography using Bio-Rad Image Analysis System and analysis using Image J software. β-actin was used as internal reference.

### Reverse transcription quantitative polymerase chain reaction (RT-qPCR)

Total RNA was extracted using Trizol reagent (15596026, Invitrogen, Carlsbad, CA, USA), and reverse transcription was performed according to the cDNA reverse transcription kit (K1622, Beijing Yaanda Biotechnology Co., Ltd., Beijing, China). The synthesized cDNA was tested by Fast SYBR Green PCR kit (Applied Biosystems) and ABI 7500 RT-PCR system (Applied Biosystems). glyceraldehyde-3-phosphate dehydrogenase (GAPDH) was used as an internal reference, and the relative expression of related genes was analyzed by 2^−ΔΔCt^ method. The primer design is shown in Table 1.

**Table 1:** Primer sequences for reverse transcription quantitative polymerase chain reaction.

	Sequences
HDAC1	Forward: CTACTACGACGGGGATGTTGG
	Reverse: GAGTCATGCGGATTCGGTGAG
NEP	Forward: AAGAAACAGCGATGGACTC
	Reverse: TTTATGCAGTCTGATGACTTG
GAPDH	Forward: AACGGATTTGGTCGTATTGGG
	Reverse: TCGCTCCTGGAAGATGGTGAT

*HDAC1* histone deacetylase 1, *NEP* neutral endopeptidase, *GAPDH* glyceraldehyde-3-phosphate dehydrogenase.

### Cell transfection

Pancreatic acinar cells AR4-2J, purchased from ATCC (USA), were cultured in Dulbecco’s Modified Eagle Medium/F12 supplemented with 20% fetal calf serum and antibiotics, and cultured in a 37 °C, 5% CO_2_ incubator (American ThermoFisher Scientific Corporation). Cells in the logarithmic phase were trypsinized, and inoculated in 6-well plates at 1 × 10^5^ cells per well. After 24 h of routine culture, cell transfection was performed according to Lipofectamine 2000 (Invitrogen) when cell confluence reached 75%. The cultured cells were transfected with the following plasmids: sh-ATF4, sh-HDAC1, sh-NEP, oe-HDAC1, oe-KFL4 and corresponding controls. Silencing plasmids (pGPU6/Neo, C02003) were purchased from Shanghai GenePharma Co., Ltd, and overexpression plasmids (pCDNA3.1-FLAG-LPA2, P1224) were purchased from Wuhan Miaoling Biotechnology Co., Ltd. (Wuhan, China) Successfully transfected cells were incubated with 500 μM Na-TC for 12 h, and then treated with L-alanyl-L-glutamine (2 mM) and cerulein to construct a cellular AP model.

### 5-ethynyl-2′-deoxyuridine (EdU) assay

The cells were seeded in a 24-well plate (three replicates were made for each group) supplemented with EdU (a concentration of 10 μmol/L) for incubation in the incubator for 2 h. The sample was fixed with PBS solution containing 4% paraformaldehyde for 15 min at room temperature, washed twice with PBS containing 3% bovine serum albumin (BSA), and incubated with PBS containing 0.5% Triton-100 at room temperature for 20 min. Next, the sample was washed by PBS containing 3% BSA, added with 100 μL of staining solution and incubated for 30 min at room temperature in the dark. Afterward, 4′,6-diamidino-2-phenylindole was added to stain the nucleus for 5 min, and then the sample was covered and observed under fluorescence microscope (model: FM-600, Shanghai Putan Optical Instrument Co., Ltd., Shanghai, China) with 6–10 randomly selected fields. The number of positive cells in each field was recorded. EdU labeling rate (%) = number of positive cells/(number of positive cells + number of negative cells) × 100%.

### Terminal deoxyribonucleotidyl transferase (TDT)-mediated 2′-deoxyuridine 5′-triphosphate-digoxigenin nick end labeling (TUNEL) assay

Apoptosis of pancreatic acinar cells was determined using the TUNEL Apoptosis Detection Kit (Beyotime Biotechnology, Shanghai, China) according to the manufacturer’s instructions. Nuclei with dark reddish-brown were identified as positive. Under a light microscope, TUNEL-positive cells were counted in 10 randomly selected high-power fields and expressed as a percentage of the total cell count (apoptosis index).

### Flow cytometry

Collected cells were centrifuged at 2000 rpm for 5 min, washed with cold PBS for two times, and suspended by 400 μL 1× Binding Buffer. Next, cell suspension was added with 5 μL AnnexinV-Fluorescein Isothiocyanate for incubation at 4 °C for 15 min in the dark, and incubated with 10 μL propidium iodide at 4 °C for 5 min in the dark. Flow cytometer (BD FACS Calibur, BD Inc., USA) was used to detect cell status within 1 h.

### Chromatin immunoprecipitation (ChIP)

Cells with confluence of 70–80% were collected and fixed with 1% formaldehyde at room temperature for 10 min to crosslink DNA and protein. After the termination by glycine, the sample was sonicated by ultrasonic treatment and centrifuged at 13,000 rpm at 4 °C, followed by incubation with RNA polymerase II, anti-IgG, anti-ATF4 (ab85049, ab59718, Abcam), anti-HDAC1 (1:100, ab59718, Abcam) at 4 °C overnight. Protein Agarose/Sepharose was used to precipitate the endogenous DNA-protein complex. After centrifugation, the supernatant was discarded, the non-specific complexes were washed, the cross-linking was de-linked at 65 °C, and the DNA fragments were purified and recovered by phenol/chloroform. RT-qPCR was used to detect the enrichment of related proteins in NEP and HDAC1 promoter regions.

### Statistics analysis

SPSS 21.0 (IBM Corp, Armonk, NY, USA) was performed for statistical analysis with *p* < 0.05 indicated statistically difference. Three independent biological experiments were replicated in each group. The comparison of the measurement data (mean standard ± deviation) between two groups were tested by independent sample *t*-test while comparison among multiple groups was analyzed by one-way analysis of variance with Tukey’s post hoc test.

## Results

### ATF4 is highly expressed in AP

**Fig. 1:**
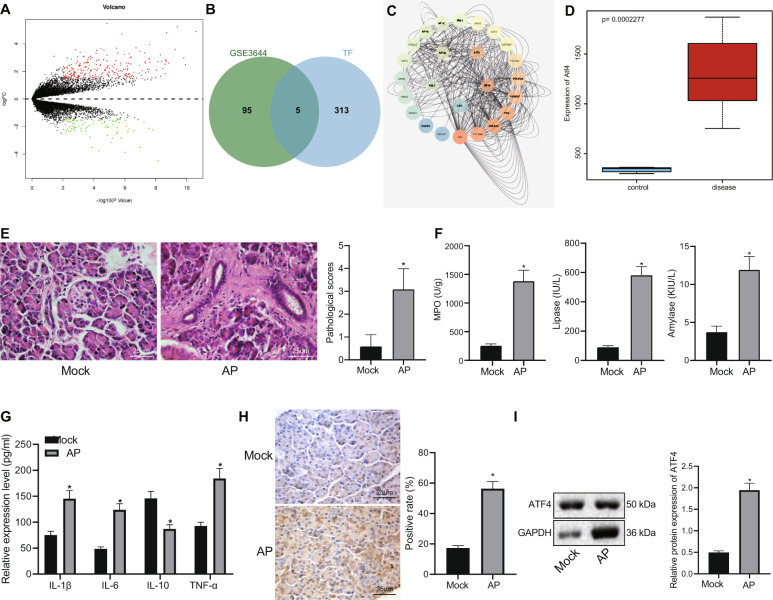
Highly expressed ATF4 is found in AP. **A** Volcano map of differentially expressed genes obtained by the difference analysis of microarray dataset GSE3644. Red dots indicate genes that are significantly upregulated, green dots indicate genes that are significantly downregulated, and black dots indicate insignificant genes. **B** Comparison results of differentially expressed genes in microarray dataset GSE3644 with transcription factors. The five intersecting genes are ATF4, HIF1A, NFYB, RBL2, and SRC. **C** The PPI network of important transcription factors and related genes constructed by GeneMANIA. The five hexagonal genes placed in the middle are the input genes, and the rounded genes are the predicted related genes. The higher the core level, the bluer the lower the core level. **D** Box line diagram (*p* = 2.28e−04) of ATF4 expression data in microarray dataset GSE3644. The blue box on the left indicates the expression of normal samples, and the red box on the right indicates the expression of the pancreatitis sample. **E** H&E staining to detect pathological conditions of pancreatic tissue. **F** Biochemical analysis to detect peroxidase, serum amylase, and serum lipase levels. **G** ELISA to detect serum IL-1β, IL-6, IL-10, and TNF-α. **H** ATF4 expression in pancreatic tissues detected by immunohistochemistry. **I** ATF4 expression in pancreatic tissue detected by western blot analysis. *p < 0.05 compared with the Mock group (normal control). The comparison of the measurement data (mean standard ± deviation) between two groups were tested by independent sample *t*-test, *n* = 12.

Based on the R language difference analysis of the GEO microarray dataset GSE3644, 335 significantly differentially expressed genes were obtained (Fig. 1A), and 318 human transcription factors were obtained from Cistrome. The intersection of significantly differentially expressed genes and transcription factors revealed five important transcription factors (Fig. 1B). Using GeneMANIA to predict genes related to important transcription factors and construct a PPI network (Fig. 1C), we found that ATF4 was the most core transcription factor with the highest total score (Table 2). A box plot of the data from the microarray dataset GSE3644 found that ATF4 was highly expressed in pancreatitis (Fig. 1D). Existing literature indicates that ATF4 is related to AP and highly expressed in pancreatitis^9^. In order to explore the expression of ATF4 in AP, we constructed an animal model of AP, and detected the pathological score of pancreatic tissues using H&E. The results showed that AP induction led to increased histopathological score (Fig. 1E). The expression of peroxidase, serum amylase, and serum lipase in each group were detected with the results indicated that AP induction led to increased expression of peroxidase, serum amylase, and serum lipase (Fig. 1F). Additionally, the levels of IL-1β, IL-6, IL-10, and TNF-α in serum were detected by ELISA which showed that AP induction resulted in elevations in IL-1β, IL-6, and TNF-α as well as reduction in IL-10 expression (Fig. 1G). The above results indicate that the model of AP was successfully constructed. Expression of ATF4 in pancreatic tissues of AP was further determined and the results revealed that ATF4 was highly expressed in the pancreatic tissues after AP induction (Fig. 1H, I). These results demonstrated that ATF4 was highly expressed in AP.

**Table 2:** The core degree of each input gene in the PPI network.

Gene	Degree	Sum weight
ATF4	45	5.872830117
HIF1A	45	5.650693503
NFYB	22	4.758269712
RBL2	18	2.967579077
SRC	8	0.714165218

Degree represents the number of interactions between genes and other genes, and sum weight represents the sum of scores of genes and other interactions.

*PPI* protein–protein interaction, *ATF4* activating transcription factor 4, *HIF1A* hypoxia inducible factor-1A, *NFYB* nuclear transcription factor Y subunit beta, *RBL2* retinoblastoma-like protein 2.

### ER-related protein is upregulated in AP

During AP, ER stress would occur in cells. Therefore, ER stress-related molecules, p-PERK and p-eIF2-α was detected in AP model and results revealed significant upregulation of CHOP, p-PERK and p-eIF2-α (Supplementary Fig. 1).

### Silencing ATF4 promotes pancreatic acinar cell proliferation and inhibits apoptosis

**Fig. 2:**
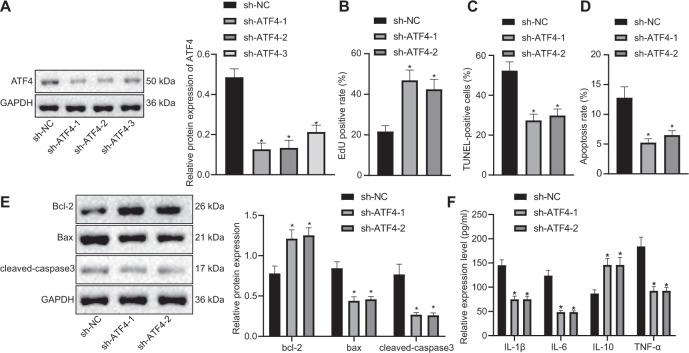
Downregulation of ATF4 promotes pancreatic acinar cell proliferation and inhibits apoptosis. **A** Western blot analysis was performed to detect ATF4 silencing efficiency. **B** EdU was applied to detect pancreatic acinar cell proliferation after sh-ATF4-1 or sh-ATF4-2 treatment. **C** TUNEL was performed to detect pancreatic acinar cell apoptosis after sh-ATF4-1 or sh-ATF4-2 treatment. **D** Flow cytometry was performed to detect pancreatic acinar cell apoptosis after sh-ATF4-1 or sh-ATF4-2 treatment. **E** Western blot analysis was performed to detect expression of Bcl-2, Bax and cleaved-caspase3. **F** ELISA was conducted to test levels of IL-1β, IL-6, TNF-α, and IL-10. *p < 0.05 compared with the sh-NC group. The comparison of the measurement data (mean standard ± deviation) among multiple groups was analyzed by one-way analysis of variance with Tukey’s post hoc test.

In order to understand the effect of ATF4 on pancreatic acinar cells in AP, we used cerulein to treat pancreatic acinar cells AR4-2J to construct an in vitro AP cell model, and the cells were treated with silenced ATF4. Western blot analysis was performed to detect ATF4 silencing efficiency and the results demonstrated that sh-ATF4-1 and sh-ATF4-2 had the best silencing efficiency (Fig. 2A), which were subsequently selected for related experiments. EdU, TUNEL and flow cytometry assays revealed that sh-ATF4-1 or sh-ATF4-2 treatment led to enhanced pancreatic acinar cell proliferation and inhibited apoptosis (Fig. 2B–D). The determination of western blot analysis and ELISA also showed that sh-ATF4-1 or sh-ATF4-2 treatment resulted in elevations in expression of Bcl-2 and IL-10 as well as reductions in expression of Bax, cleaved-caspase3, IL-1β, IL-6, and TNF-α (Fig. 2E, F). The above results indicated that silencing ATF4 promotes pancreatic acinar cell proliferation and inhibits apoptosis.

### ATF4 binds to the HDAC1 promoter and upregulates its expression in AP

**Fig. 3:**
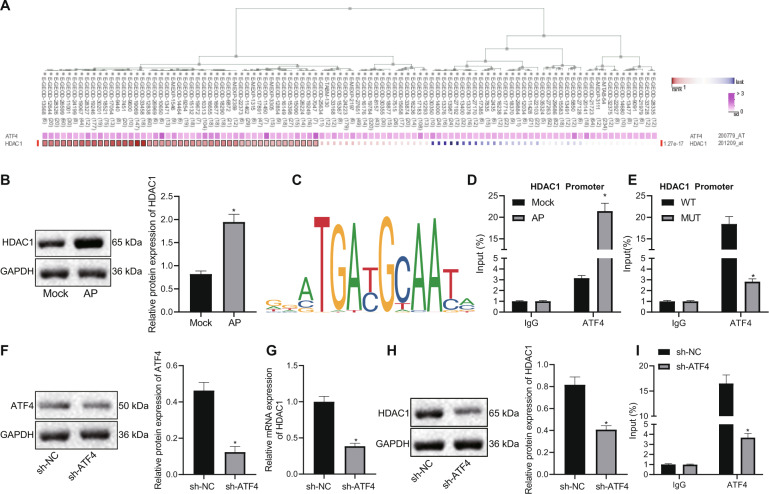
ATF4 binds to the HDAC1 promoter and upregulates its expression in AP. **A** Co-expression analysis with MEM to determine the co-expression relationship between ATF4 and HDAC1 (*p* = 1.27e−17). **B** Expression of HDAC1 in an AP animal model detected by western blot analysis. **C** The binding site of ATF4 and HDAC1 promoter obtained through JASPAR. **D** ChIP was used to detect the enrichment of ATF4 in the HDAC1 promoter. **E** After mutation, ChIP was applied to detect the enrichment of ATF4 in the HDAC1 promoter. **F** Western blot analysis was used to detect the ATF4 expression in each group. **G** RT-qPCR was applied to detect the expression of HDAC1. **H** Western blot analysis was applied to detect the expression of HDAC1. **I** ChIP was used to detect the enrichment of ATF4 in the HDAC1 promoter. *p < 0.05 compared with the Mock, WT or sh-NC group. The comparison of the measurement data (mean standard±deviation) between two groups were tested by independent sample *t*-test.

Previous study has shown that ATF4 can promote HDAC1 expression through the HDAC1 promoter^10^. We used co-expression analysis with MEM to determine the co-expression relationship between ATF4 and HDAC1 (*p* = 1.27e−17) (Fig. 3A). In order to understand the relationship between ATF4 and HDAC1, we detected the expression of HDAC1 in an AP animal model by western blot analysis, and the results showed that HDAC1 was highly expressed in AP (Fig. 3B). We obtained the binding site of ATF4 and HDAC1 promoter through JASPAR (Fig. 3C). Then we used ChIP to detect the enrichment of ATF4 in the HDAC1 promoter in AP, and the results showed that ATF4 enrichment in the HDAC1 promoter region was significantly increased after AP induction (Fig. 3D). We mutated the binding site and used ChIP to detect the enrichment of ATF4 in the HDAC1 promoter. The results indicated that compared with the wild type (WT) group, the AUT4 enrichment in the HDAC1 promoter region was significantly reduced in the mutant (MUT) group (Fig. 3E). After we silenced ATF4, western blot analysis was used to detect the ATF4 expression in each group, and the results revealed successful silencing of ATF4 expression (Fig. 3F). In addition, RT-qPCR and western blot analysis were applied to detect the expression of HDAC1 in each group, and the results showed that the mRNA and protein expression of HDAC1 in the sh-ATF4 group was significantly reduced (Fig. 3G, H). ChIP was used to detect the enrichment of ATF4 in the HDAC1 promoter. The results demonstrated that the enrichment of ATF4 in the HDAC1 promoter of the sh-ATF4 group was significantly reduced (Fig. 3I). These results concluded that ATF4 could bind to the HDAC1 promoter and upregulate its expression in AP.

### Silencing ATF4 inhibits apoptosis of pancreatic acinar cells by downregulation of HDAC1 expression

**Fig. 4:**
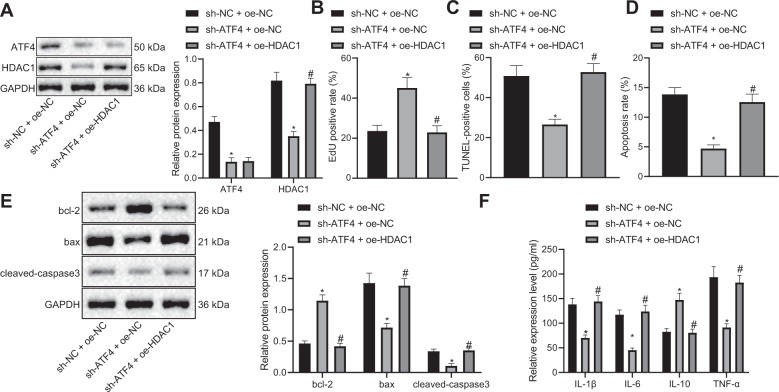
Silencing ATF4 inhibits apoptosis of pancreatic acinar cells by downregulation of HDAC1 expression. **A** Western blot analysis was performed to detect ATF4 and HDAC1. **B** EdU was applied to detect pancreatic acinar cell proliferation after sh-ATF4 or oe-HDAC1 treatment. **C** TUNEL was performed to detect pancreatic acinar cell apoptosis after sh-ATF4 or oe-HDAC1 treatment. **D** Flow cytometry was performed to detect pancreatic acinar cell apoptosis after sh-ATF4 or oe-HDAC1 treatment. **E** Western blot analysis was performed to detect expression of Bcl-2, Bax and cleaved-caspase3. **F** ELISA was conducted to test levels of IL-1β, IL-6, TNF-α, and IL-10. *p < 0.05 compared with the sh-NC + oe-NC group; ^#^*p* < 0.05 compared with the sh-ATF4 + oe-NC group. The comparison of the measurement data (mean standard ± deviation) among multiple groups was analyzed by one-way analysis of variance with Tukey’s post hoc test.

In order to understand the effect of ATF4 and HDAC1 on pancreatic acinar cells in AP, we used cerulein to treat pancreatic acinar cells AR4-2J to construct an AP cell model in vitro, and cells were then treated with silenced ATF4 or overexpressed HDAC1. After treatment, the expressions of ATF4 and HDAC1 in each group were detected by western blot analysis, and the results showed that silenced sh-ATF4 treatment led to reduced expressions of ATF4 and HDAC1 while there was no significant difference in ATF4 expression but increased HDAC1 after oe-HDAC1 treatment (Fig. 4A). EdU, TUNEL, and flow cytometry assays revealed that sh-ATF4 treatment led to enhanced pancreatic acinar cell proliferation and inhibited apoptosis which could be reversed by oe-HDAC1 treatment (Fig. 4B–D). The determination of Western blot analysis and ELISA also showed that sh-ATF4 treatment resulted in elevations in expression of Bcl-2 and IL-10 as well as reductions in expression of Bax, cleaved-caspase3, IL-1β, IL-6, and TNF-α which could be reversed by oe-HDAC1 treatment (Fig. 4E, F). The above results indicated that silencing ATF4 promotes pancreatic acinar cell proliferation and inhibits apoptosis through suppression of HDAC1.

### HDAC1 promotes KLF4 expression by inhibiting NEP expression

**Fig. 5:**
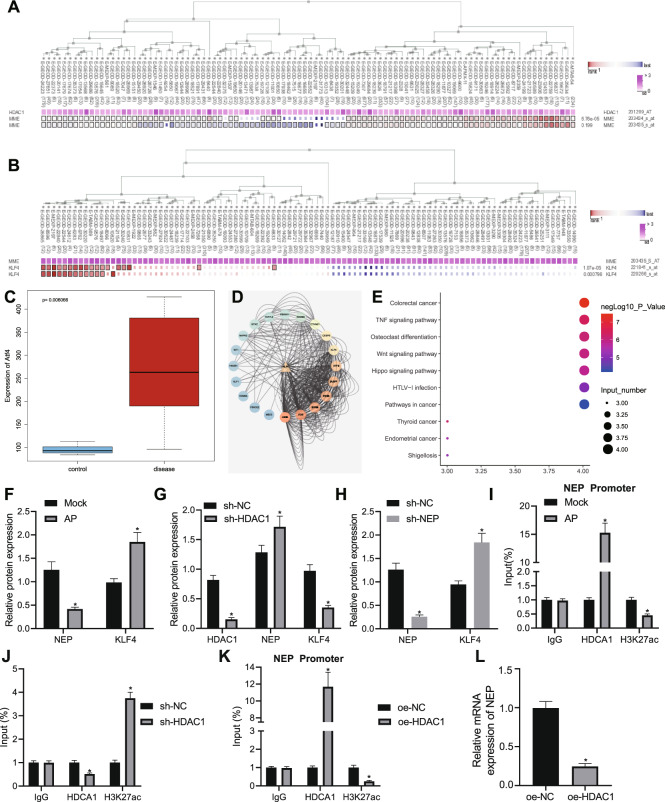
HDAC1 promotes KLF4 expression by inhibiting NEP expression. **A** Co-expression analysis with MEM to determine the co-expression relationship between HDAC1 and NEP (*p* = 6.76e−05). **B** Co-expression analysis with MEM to determine the co-expression relationship between NEP and KLF4 (*p* = 6.76e−05). **C** A box plot from the expression data of the microarray dataset GSE3644 to determine expression of KLF4. The blue box on the left represents the expression of normal samples, and the red box on the right represents the expression of pancreatitis samples. **D** GeneMANIA predicted KLF4 related genes and constructed a PPI network diagram. The triangle gene in the middle is KLF4, and the outer circle gene is the predicted related gene. The redder the color of the gene, the higher the core degree, and vice versa the bluer the core, the lower the degree. **E** KOBAS was performed for KEGG enrichment analysis of KLF4 and its related genes. The vertical axis represents the enriched pathway. The horizontal axis and the size of the bubble represent the number of genes enriched on the pathway. Significance-log*P* value, the greater the significance, the redder the bubbles, and the smaller the significance, the bluer the bubbles. **F** Western blot analysis to detect the expression of NEP and KLF4 in AP. **G** Western blot analysis to detect the expression of HDAC1, NEP, and KLF4 in AP after silencing NEP. **H** Western blot analysis to detect the expression of NEP and KLF4 in AP. **I** ChIP detected the enrichment of HDAC1 and H3K27ac in the NEP promoter of each group. **J** ChIP detected the enrichment of HDAC1 and H3K27ac in the NEP promoter of each group after silencing NEP. **K** ChIP detected the enrichment of HDAC1 and H3K27ac in the NEP promoter of each group. **L** RT-qPCR detected HDAC1 mRNA expression. *p < 0.05 compared with the Mock, WT or sh-NC group. The comparison of the measurement data (mean standard ± deviation) between two groups were tested by independent sample *t*-test.

Some literature show that HDAC1 binds to the NEP promoter and inhibits the transcription of NEP by removing H3K27ac modification^14^. NEP is poorly expressed in caerulein-induced AP and overexpressed NEP can inhibit AP^16^. In addition, NEP can inhibit the expression of KLF4^17^. Co-expression was conducted through MEM found that HDAC1 and NEP have a significant co-expression relationship (*p* = 6.76e−05) (Fig. 5A), and there is also a significant co-expression relationship between NEP and KLF4 (*p* = 1.07e−05) (Fig. 5B). A box plot was drawn from the expression data of the microarray dataset GSE3644 to determine the high expression of KLF4 in AP (Fig. 5C). GeneMANIA was used to predict the KLF4 related genes and construct a PPI network (Fig. 5D). KEGG enrichment analysis was performed in KLF4 and related genes through KOBAS, the results showed that KLF4 was correlated with AP (Fig. 5E). In order to understand the regulatory relationship of HDAC1 on NEP-KLF4, we used Western blot analysis to detect the expression of NEP and KLF4 in AP. The results revealed that NEP expression was low and KLF4 expression was high in AP (Fig. 5F). After silencing HDAC1, decreased HDAC1 and KLF4 expression but increased NEP expression were found (Fig. 5G). After silencing NEP, decreased NEP expression but increased KLF4 expression were found (Fig. 5H). ChIP was used to detect the enrichment of HDAC1 in the NEP promoter. The results showed that HDAC1 enrichment in NEP promoter significantly increased and H3K27ac enrichment decreased significantly in AP (Fig. 5I). After silencing HDAC1, HDAC1 enrichment significantly reduced in the NEP promoter region, and H3K27ac enrichment significantly increased (Fig. 5J). ChIP demonstrated that oe-HDAC1 treatment led to increased HDAC1 enrichment but decreased H3K27ac enrichment (Fig. 5K). Moreover, RT-qPCR determination demonstrated that oe-HDAC1 treatment resulted in increased HDAC1 mRNA (Fig. 5L). The above results clarified that HDAC1 promotes KLF4 expression by inhibiting NEP expression.

### Silencing HDAC1 inhibits KLF4 expression by upregulating NEP expression thus promoting pancreatic acinar cell proliferation

**Fig. 6:**
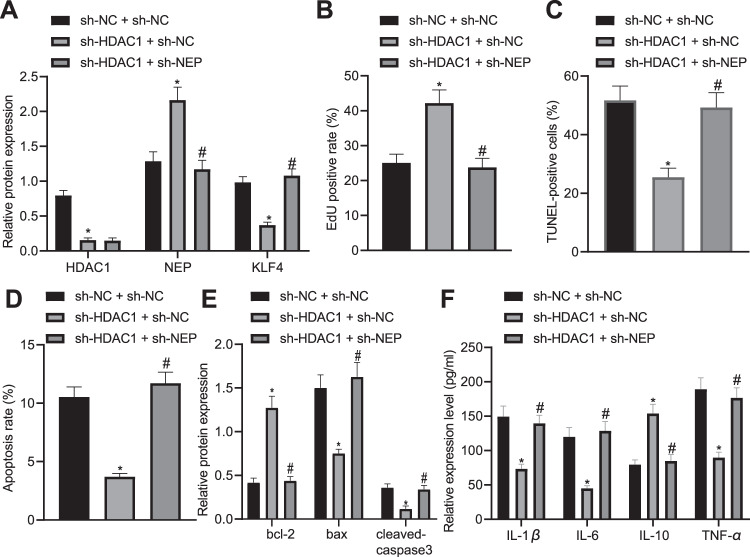
Downregulation of HDAC1 inhibits KLF4 expression by upregulating NEP expression thus promoting pancreatic acinar cell proliferation. **A** Western blot analysis was performed to detect HDAC1, NEP, and ATF4. **B** EdU was applied to detect pancreatic acinar cell proliferation after sh-HDAC1 or sh-NEP treatment. **C** TUNEL was performed to detect pancreatic acinar cell apoptosis after sh-HDAC1 or sh-NEP treatment. **D** Flow cytometry was performed to detect pancreatic acinar cell apoptosis after sh-HDAC1 or sh-NEP treatment. **E** Western blot analysis was performed to detect expression of Bcl-2, Bax, and cleaved-caspase3. **F** ELISA was conducted to test levels of IL-1β, IL-6, TNF-α, and IL-10. *p < 0.05 compared with the sh-NC + oe-NC group; ^#^*p* < 0.05 com*p*ared with the sh-HDAC1 + oe-NC group. The comparison of the measurement data (mean standard±deviation) among multiple groups was analyzed by one-way analysis of variance with Tukey’s post hoc test.

In order to understand the effect of HDAC1, NEP, and ATF4 on pancreatic acinar cells in AP, we used cerulein to treat pancreatic acinar cells AR4-2J to construct an AP cell model in vitro, and cells were then treated with silenced NEP or silenced HDAC1. After treatment, the expressions of HDAC1, NEP and KLF4 in each group were detected by Western blot analysis, and the results showed that sh-HDAC1 treatment led to reductions in HDAC1 and KLF4 but elevation in NEP expression while there was no significant difference in HDAC1 expression but increased KLF4 and decreased NEP after sh-NEP treatment (Fig. 6A). EdU, TUNEL and flow cytometry assays revealed that sh-HDAC1 treatment led to enhanced pancreatic acinar cell proliferation and inhibited apoptosis which could be reversed by sh-NEP treatment (Fig. 6B–D). The determination of Western blot analysis and ELISA also showed that sh-HDAC1 treatment resulted in elevations in expression of Bcl-2 and IL-10 as well as reductions in expression of Bax, cleaved-caspase3, IL-1β, IL-6, and TNF-α, which could be reversed by sh-NEP treatment (Fig. 6E, F). The above results indicated that silencing HDAC1 inhibits KLF4 expression by upregulating NEP expression thus promoting pancreatic acinar cell proliferation.

### Silencing ATF4 regulates NEP-KLF4 expression by inhibiting HDAC1 thus promoting pancreatic acinar cell proliferation

**Fig. 7:**
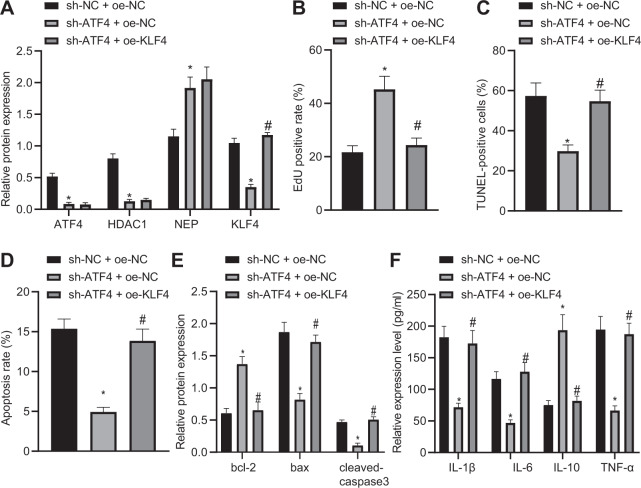
Downregulation of ATF4 promotes pancreatic acinar cell proliferation via regulation of NEP-KLF4 expression by inhibiting HDAC1. **A** Western blot analysis was performed to detect ATF4, HDAC1, NEP, and KLF4. **B** EdU was applied to detect pancreatic acinar cell proliferation after sh-ATF4 or oe-KLF4 treatment. **C** TUNEL was performed to detect pancreatic acinar cell apoptosis after sh-ATF4 or oe-KLF4 treatment. **D** Flow cytometry was performed to detect pancreatic acinar cell apoptosis after sh-ATF4 or oe-KLF4 treatment. **E** Western blot analysis was performed to detect expression of Bcl-2, Bax, and cleaved-caspase3. **F** ELISA was conducted to test levels of IL-1β, IL-6, TNF-α, and IL-10. *p < 0.05 compared with the sh-NC + oe-NC group; ^#^*p* < 0.05 com*p*ared with the sh-ATF4 + oe-NC group. The comparison of the measurement data (mean standard ± deviation) among multiple groups was analyzed by one-way analysis of variance with Tukey’s post hoc test.

Additionally, the effect of ATF4, HDAC1, NEP, and KLF4 on pancreatic acinar cells in AP was investigated after establishment of an AP cell model, and cells were then treated with silenced ATF4 or silenced KLF4. After treatment, the expressions of ATF4, HDAC1, NEP, and KLF4 in each group were detected by western blot analysis, and the results showed that sh-ATF4 treatment led to increased NEP expression but decreased ATF4, HDAC1, and KLF4 while there was no significant change in ATF4, HDAC1, and NEP but increased KLF4 after oe-KLF4 treatment (Fig. 7A). EdU, TUNEL, and flow cytometry assays revealed that sh-ATF4 treatment led to enhanced pancreatic acinar cell proliferation and inhibited apoptosis which could be reversed by oe-KLF4 treatment (Fig. 7B–D). The determination of western blot analysis and ELISA also showed that sh-ATF4 treatment resulted in elevations in expression of Bcl-2 and IL-10 as well as reductions in expression of Bax, cleaved-caspase3, IL-1β, IL-6, and TNF-α, which could be reversed by oe-KLF4 treatment (Fig. 7E, F). The above results indicated that silencing ATF4 promotes pancreatic acinar cell proliferation via regulation of NEP-KLF4 expression by inhibiting HDAC1.

### Downregulation of ATF4 suppresses AP via regulation of NEP-KLF4 expression by inhibiting HDAC1

**Fig. 8:**
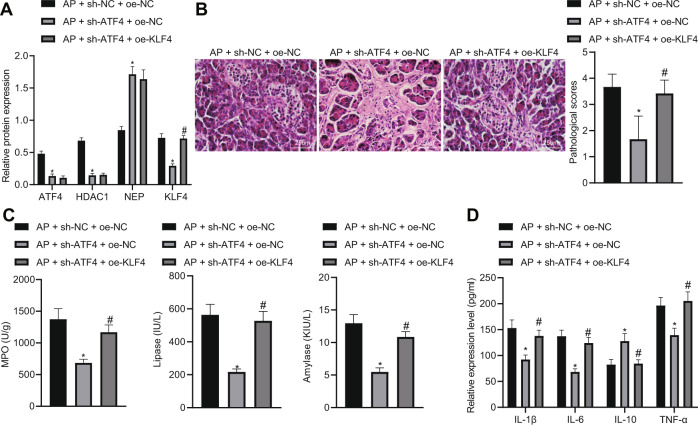
Downregulation of ATF4 suppresses AP via regulation of NEP-KLF4 expression by inhibiting HDAC1. **A** Western blot analysis was performed to detect ATF4, HDAC1, NEP, and KLF4. **B** HE staining to detect pathological conditions of pancreatic tissue after sh-ATF4 or oe-KLF4 treatment in AP mice. **C** Biochemical analysis to detect peroxidase, serum amylase, and serum lipase levels after sh-ATF4 or oe-KLF4 treatment in AP mice. **D** ELISA to detect serum IL-1β, IL-6, IL-10, and TNF-α in AP mice; *p < 0.05 compared with the AP + sh-NC + oe-NC group; ^#^*p* < 0.05 com*p*ared with the AP + sh-ATF4 + oe-NC group. The comparison of the measurement data (mean standard ± deviation) among multiple groups was analyzed by one-way analysis of variance with Tukey’s post hoc test.

Furthermore, the regulatory role of ATF4 in animal model was also explored. After different treatments, the expressions of ATF4, HDAC1, NEP, and KLF4 in each group were detected by western blot analysis, and the results showed that AP mice with sh-ATF4 treatment led to increased NEP expression but decreased ATF4, HDAC1, and KLF4 while there was no significant change in ATF4, HDAC1 and NEP but increased KLF4 after oe-KLF4 treatment (Fig. 8A). The pathological score of pancreatic tissues in AP mice with different treatment was determined using H&E. The results showed that AP mice with sh-ATF4 treatment led to reduced histopathological score while AP mice with both sh-ATF4 and oe-KLF4 treatment showed increased histopathological score (Fig. 8B). The expression of peroxidase, serum amylase, and serum lipase in each group were detected with the results indicated that AP mice with sh-ATF4 treatment led to reduced expression of peroxidase, serum amylase, and serum lipase while AP mice with both sh-ATF4 and oe-KLF4 treatment showed increased expression of peroxidase, serum amylase, and serum lipase (Fig. 8C). Moreover, the levels of IL-1β, IL-6, IL-10 and TNF-α in serum were detected by ELISA which showed that AP mice with sh-ATF4 treatment resulted in reductions in IL-1β, IL-6, and TNF-α as well as elevation in IL-10 expression while AP mice with both sh-ATF4 and oe-KLF4 treatment led to elevations in IL-1β, IL-6 and TNF-α as well as reduction in IL-10 expression (Fig. 8D). The above findings demonstrated that silencing ATF4 suppresses AP via regulation of NEP-KLF4 expression by inhibiting HDAC1.

## Discussion

Currently, increasing attention has been paid to basic study regarding AP, including immunomodulatory therapy and mesenchymal stem cell-based therapy^20^. Notably, implication of miRNAs in AP has been deciphered^21^. However, investigation with regard to the role of functional gene signaling pathway in AP is limited. More importantly, ER has been identified to be activated in AP^22^. Mitochondrial dysfunction has been recognized to induce ER stress and play a negative role in AP progression^23^. Disorders of the acinar cells involve in the induction of pancreatitis through mediating inflammatory response^4^. Our study paid the main attention to the roles of ER stress-related ATF4, HDAC1, NEP, and KLF4 in the growth, apoptosis, and inflammation in pancreatic acinar cells. The obtained results demonstrated that ATF4 silencing contributed to protection against AP via inhibiting the recruitment of HDAC1 in the NEP promoter and downregulating KLF4, providing a novel insight into developing potential therapeutic strategy for AP.

The first major observation was the aberrant high expression of ATF4 in the pancreatic tissues of AP mouse model. Further knockdown experiment provided evidence for the conclusion that ATF4 loss-of-function resulted in potentiated proliferative capability and suppressed apoptosis of pancreatic acinar cells, which was also supported by an increase of Bcl-2 protein and reductions of Bax and cleaved-caspase3 proteins. ATF4 is one of unfolded protein response components involved in the ER stress and may also induce cell death through the modulation of mitochondrial apoptosis-related Bcl-2 family proteins^24^. In a previous study, ATF4 knockout was associated with the apoptosis of pancreatic acinar cells^25^. Also, ATF4 activation may induce cell apoptosis in exocrine pancreas^26^. In addition to the pro-proliferative and anti-apoptotic functions achieved by ATF4 knockdown, our experiments further identified an anti-inflammatory potential, corresponding to reduced levels of pro-inflammatory proteins IL-1β, IL-6, and TNF-α and increased level of anti-inflammatory protein IL-10. ATF4 exerts manifold role in the development of pathologies where ATF4 controls many cellular signaling pathways, such as autophagy, oxidative stress, and inflammatory response. For instance, the pro-inflammatory effect of ATF4 through activation of IL-6 has been illustrated in the investigation conducted by Iwasaki et al.^27^. Consistently, specific siRNA-induced silencing of ATF4 impaired NF-κB activation and inhibited the production of pro-inflammatory cytokines^28^. In addition, downregulation of ATF4 was witnessed to ameliorate retinal inflammation in a mouse diabetic model^29^. At last, targeting ATF4 exhibited protective effect against AP-induced tissue damage and suppressive effect on the release of pro-inflammatory proteins in the mouse model. Those data contributed to a theoretical basis of the ATF4-targeted therapy for the treatment of AP.

Apart from the above-mentioned function of ATF4, we additionally analyzed the molecular mechanisms involved in this function. The co-expression of HDAC1 and ATF4 was defined by MEM analysis, while ChIP assay further confirmed the binding of ATF4 in the HDAC1 promoter. It has been reported that ATF4 transcriptionally elevates the HDAC1 expression^10^, which is also demonstrated in our study. In addition, downregulation of HDAC1 was found to be responsible for the suppression in apoptosis of pancreatic acinar cells and inflammation mediated by ATF4 silencing. Loss of HDAC1 could prolong nuclear IL-1β-triggered phosphorylation of nuclear factor kappa-B and control the levels of IL-1β-dependent inflammatory genes in intestinal epithelial cells^30^. This role of HDAC1 in inflammation has also been elaborated in another study that HDAC1 inhibition blocks the suppression of pro-inflammatory responses^31^. HDAC1, a histone deacetylase, was found to reduce H3K27ac in the NEP promoter and hence downregulate NEP expression. NEP deficiency resulted in uncontrolled inflammation^32^. NEP functioned as a repressor of pancreatic elastase-caused lung injury possibly through downregulation of pro-inflammatory proteins^33,34^. Acute inhibition of NEP increased Substance P level in caerulein-induced AP, and consequently augmented inflammation in the pancreas and the related lung damage^16^. The results of rescue experiments demonstrated that NEP knockdown reversed the pro-proliferative, anti-apoptotic and anti-inflammatory effects of HDAC1 knockdown on pancreatic acinar cells, suggesting that HDAC1 knockdown protected pancreatic acinar cells from apoptosis and inflammation through increasing NEP. KLF4 was determined to be a downstream gene of NEP, which was consistent with the previous study^17^. KLF4 was proposed to be a promoter of colonic inflammation^35^. Also, KLF4 has been reported to reduce cell viability by arresting cell cycle in G1 phase and increase cell sensitivity to apoptosis by downregulating Bcl-2^36^. Additionally, overexpressed KLF4 has been deciphered to activate p53-mediated cell apoptosis and KLF4 antagonist can activate the JAK-STAT3 signaling pathway in contribution to cell growth^37^. Moreover, KLF4 can mediate cell apoptosis by activating noncoding RNA metastasis-associated lung adenocarcinoma transcript 1^38^. However, its role in AP has been rarely mentioned. The loss-of-function and rescue experiments provided evidence for the idea that KLF4 overexpression reversed the effects of ATF4 knockdown on pancreatic acinar cells. Aforementioned evidence suggested that ATF4 mediated the HDAC1/NEP/KLF4 axis by which ATF4 participated in the prevention of AP.

In summary, our data unraveled the anti-inflammatory function of specific ATF4 shRNA, which was evidenced in the in vivo mouse model of AP. An inflammation-related pathway HDAC1/NEP/KLF4 was proposed in this study. Those findings, therefore, highlight that those genes exert promise as therapeutic targets against AP. Further clinical trials are of great concern to demonstrate the practicability of those targets in the future.

## Supplementary information

Legend of Supplementary Figure 1

figure S1

## Data Availability

The datasets generated and/or analyzed during the current study are available from the corresponding author on reasonable request.
