# Supplementary material for: ATF4-mediated histone deacetylase HDAC1 promotes the progression of acute pancreatitis
Source: Cell Death Dis. 2021 Jan 4;12(1):5. doi: 10.1038/s41419-020-03296-x (PMC7791124; doi:10.1038/s41419-020-03296-x)
Supplement: Supplementary file 1 — Legend of Supplementary Figure 1 [file 41419_2020_3296_MOESM1_ESM.docx]

**Supplementary Figure 1** Upregulation of ER stress-related proteins are identified in AP. A, p-PERK expression in AP normalized to GAPDH determined by Western blot analysis; B, p-eIF2-α expression in AP normalized to GAPDH determined by Western blot analysis; C, ATF4 expression in AP normalized to GAPDH determined by Western blot analysis; D, CHOP expression in AP normalized to GAPDH determined by Western blot analysis; ** *p* < 0.01 compared with the Mock group. The comparison of the measurement data (mean standard ± deviation) between two groups were tested by independent sample *t*-test. n = 12.
